# 

**Published:** 1919-02

**Authors:** 


### CONTENTS FOR THE FEBRUARY ISSUE

EDITORIALS.

Just the Things (Poem) .......... 259

The Visiting Doctor .......... 260

Open Air and Baths .......... 262

ORIGINAL ARTICLES.

The Biochemistry of Pellagra. E. M. Perdue, M. D. .......... 263

Pellagra and War Diet. J. R. Lowrey, M. D .......... 277

Eating for Efficiency. M. M. Carrick, M. D .......... 280

Miss Lathrop Points to New Zealand as Model .......... 281

News Items .......... 282

Army News .......... 283

Book Reviews .......... 285

Publisher's Department .......... 286

Old Jokes .......... 292

